# Depressive symptoms and smoking among young Turkish and Moroccan ethnic minority groups in the Netherlands: a cross-sectional study

**DOI:** 10.1186/1747-597X-6-5

**Published:** 2011-03-17

**Authors:** Ceren Z Acartürk, Vera Nierkens, Charles Agyemang, Karien Stronks

**Affiliations:** 1 Department of Public Health, Academic Medical Center, University of Amsterdam P.O. Box 22660, 1100 DD Amsterdam, The Netherlands

## Abstract

**Background:**

Although evidence indicates a strong association between depressive symptoms and smoking among host and migrant adults, less is known about this relationship among young ethnic minority groups in Europe. This paper aims to assess the relationship between depressive symptoms and smoking among young Turkish and Moroccan migrants in the Netherlands.

**Methods:**

Multiple logistic regression analyses was used to analyze cross-sectional data from a sample of 364 Turkish and Moroccan migrants aged 15 to 24 years. The Center for Epidemiologic Studies Depression Scale (CES-D) was used to measure the presence of clinically significant depressive symptoms. Smoking behavior was measured by a number of questions.

**Results:**

Of the respondents, 22% were smokers and 33% had depressive symptoms. The prevalence of depressive symptoms was significantly higher in smokers (42.9%) than in nonsmokers (29.5%). Respondents with depressive symptoms had increased odds of smoking even after adjusting for socioeconomic and cultural factors (OR = 2.68, 95% CI = 1.45-4.97).

**Conclusions:**

Depressive symptoms were significantly associated with smoking behavior in young Turkish and Moroccan migrants. In addition to other acknowledged factors, depressive symptoms should also be considered in relation to the smoking behavior of this group. Intervention programs for smoking behavior should take depressive symptoms into account for young Turkish and Moroccan migrants.

## Introduction

Smoking behavior is associated with mental health disorders, particularly depressive symptoms [1-4]. Smoking prevalence is higher among individuals with depressive symptoms than it is among those with no depressive symptoms [5]. Different causal pathways between depressive symptoms and smoking have been reported. Previous studies have found the use of nicotine to increase positive affect and/or decrease negative affect [6,7]. On the other hand, there is evidence showing that smoking triggers first-ever incidence of depression [8]. Some studies direct attention to a third independent factor: genetic predisposition to both smoking and depressive symptoms [9].

An important target group for preventing smoking-related diseases is young adults, who are at risk of smoking and developing tobacco-related diseases [10,11]. Young adulthood has been seen as an important transition period from occasional smoking to regular smoking, and smoking behavior is probably established during this time [12]. Young adults also act as role models for adolescents. Moreover, although they attempt to quit smoking more often than older adults, they are less likely to succeed. Thus, it is important to develop smoking intervention programs specific to young adults [13].

In addition, young adults not only have high smoking rates, they also have a high prevalence of mental health disorders. A population-based study in the Netherlands indicated that the highest prevalence rates of substance use disorders are among 18- to 25-year-olds [14]. Data from population-based studies in the US and Australia supported the highest comorbidity rates between smoking and mental health disorders in the young adult group [15]. Findings in the Netherlands showed that one out of five young adults have substance use disorders, and that they have the highest risk of having had two or more mental health disorders in the previous twelve months [14]. Although depressive disorders are associated with smoking initiation and daily smoking, it also negatively affects smoking cessation and relapse [16]. Most of the knowledge related to depressive disorders and smoking comes from adult samples.

Within the young adult group, young ethnic minorities may have a high risk of both smoking and depressive symptoms [17]. There are indications that some adult ethnic minority groups have high levels of depressive symptoms and high smoking prevalence rates compared to the host population. A recent population-based study in the Netherlands found a higher prevalence of depression among Turkish-Dutch (14.9%) and Moroccan-Dutch (6.6%) people compared to their native Dutch counterparts (4.4%) [18]. Little is known about the relationship between depressive symptoms and smoking among young ethnic minority and migrant groups in Europe. Many of the studies on ethnic differences in the association between smoking and depressive symptoms have been conducted in American and European adult samples. Young adults from ethnic minorities are of particular interest in this respect. They appear more likely to develop risk behaviors and mental health problems. These risks could be related to the high levels of acculturative stress associated with prejudice, discrimination, and/or the dual pressure of maintaining their culture of origin as well as adapting to the culture of majority [19].

Although both smoking and depressive symptoms may be highly prevalent among ethnic minorities during young adulthood, there is limited knowledge about the association between depressive symptoms and smoking in young adult migrants. Young ethnic minorities with acculturative stress who are in the critical years of young adulthood may be more vulnerable to depressive symptoms and smoking. It is important to study the association between depressive symptoms and smoking in this group to better understand the mechanisms, which will contribute to the development of culturally sensitive intervention programs. For this reason, in the present study we aimed to investigate the association between depressive symptoms and smoking behavior in young Turkish and Moroccan ethnic minority groups aged 15 to 24 years in the Netherlands. To arrive at a comprehensive overview, we used the same model to examine the effects of socioeconomic status and cultural factors.

## Methods

### Data

We analyzed the cross-sectional data from the LASER study (Lifestyle in Amsterdam: a Study among Ethnic gRoups). These data on the health-related risk factors and their determinants among Turkish-Dutch and Moroccan-Dutch adolescents and young adults were collected between 2003 and 2004. The study was approved by the Medical Ethics Committee of the Academic Medical Center (AMC) in Amsterdam, the Netherlands. Individuals aged between 10 and 30 years who were either born in Turkey or Morocco or had at least one parent who was born in Turkey or Morocco were randomly sampled through the Amsterdam population registry. Trained interviewers who were matched according to ethnic background and gender collected the data through face-to-face interviews. Written informed consent was obtained from each participant. Close response rates were observed: 57.0% for the Turkish-Dutch group and 55.0% for the Moroccan-Dutch group. The study population was representative of the total Turkish and Moroccan female population in Amsterdam with regard to gender, generational status, and city district. A more detailed description of the LASER study can be found elsewhere [20].

### Respondents

In the present study, the sample consisted of 364 adolescents and young adults (43.7% Moroccan-Dutch and 56.3% Turkish-Dutch) aged between 15 and 24 years. The sample was comprised of 179 females (51% Turkish-Dutch) and 185 males (62% Turkish-Dutch). Only those in this age group completed the Center for Epidemiologic Studies Depression Scale (CES-D) [21]. Respondents who were born in Turkey or Morocco were classified as first-generation, while those who born in the Netherlands with at least one parent born in either Turkey or Morocco were classified as second-generation.

### Measures

#### Smoking behavior

Nicotine use was measured with the smoking uptake continuum theory of Flay and colleagues [22,23]. Self-perception of smoking behavior was assessed with a number of questions. Because of the age of the participants (15 to 30 years), we used a broad definition of being a smoker. We included triers and experimenters who smoked monthly or tried only occasionally. Nine statements were provided, such as "I smoke at least one cigarette a day," "I try smoking once in a while," and "I have never smoked, not even a single puff." Moreover, participants who had smoked more than 100 cigarettes in their life were also coded as smokers.

#### Background variables

A questionnaire regarding information on age (in years), gender, marital status (married/cohabiting vs not married/cohabiting), labor market position (employed vs student), and generational status (first- vs second-generation) was administered Participants' education level was categorized as "low" (no education, primary school education, or lower-level vocational training) and "middle to high" (intermediate-level vocational training, higher secondary school levels, professional education, and university).

#### Cultural factors

Cultural factors consisted of three indicators. First, *ethnicity *(Turkish-Dutch or Moroccan-Dutch) was assessed by asking participants if they or at least one of their parents were born in Turkey or Morocco.

In addition, the *perceived importance of religion *was measured with one item, which ranged from very important to not important at all. In the four-point scale, participants who indicated that religion was very important (score 4) were categorized as one group, while the second group included the participants who indicated that religion was not important at all to moderately important (scores 1-3, low). Because the majority of the participants indicated that religion was very important to them, we put them into a separate group.

Finally, we based our definition of *acculturation *on Berry's approach (which considers a person's position in terms of his or her orientation towards the majority culture vs their culture of origin, and social contacts with the host population vs contacts with people from their culture of origin) [24]. The indicators of acculturation had two components. First, *cultural orientation *was measured by 10 items on language use with family members and friends, use of media, difficulties with reading Dutch, shopping preferences, and emancipation as examples of Western norms and values. The scale was constructed using principal component analysis and reliability analysis (alpha = 0.64). Second, *social contacts *with native Dutch people during leisure time were measured by three questions (alpha = 0.84). For both scales, the scores on the items were added up and the tertiles were constructed. The subjects in the upper tertile were categorized as having a high cultural orientation towards Dutch culture or a high level of social contacts with ethnic Dutch people. Those in the remaining two tertiles were categorized as having a low cultural orientation or a low level of social contacts [20].

#### Depression

**C**linically significant depressive symptoms were measured with the Center for Epidemiologic Studies Depression Scale (CES-D) [21], which is a validated scale. The CES-D, which was developed to assess depressive symptoms in the general population, is also applicable to adolescents and young adults [25]. The 20 statements referring to depressed affect, positive affect, somatic and psychomotor retardation, and interpersonal symptoms were rated on a four-point Likert scale. After the four items were reverse-coded, scores on all 20 items were added up to get a total score. A higher score indicates higher depressive symptomatology. Using the CES-D guidelines, we classified respondents with a score of 16 or higher as having depressive symptoms, and those with a lower score as having no depressive symptoms. The missing values up to four were replaced with the respondents' mean score.

### Analyses

Univariable logistic regression (adjusted for age) was examined to determine the relationships between the background variables, cultural factors, depressive symptoms, and the outcome measure (smoking behavior). Then, we estimated the odds ratio (OR) and corresponding 95% confidence interval (CI) for depressive symptoms with multivariable logistic regression, while adjusting for factors that appeared to be associated with smoking (age, gender, labor market position, ethnicity, and religion). Because the interaction effect of gender on the association between depressive symptoms and smoking was not significant, the sample was taken as whole instead of comparing males and females. All statistical operations were conducted using SPSS version 16.0 (SPSS Inc., Chicago).

## Results

### Characteristics of the sample

The characteristics of the sample are presented in Table 1. The prevalence of current smoking behavior was 22.3%. Thirty-three percent had depressive symptoms. The mean age of the sample was 18.8 years (SD 2.7). The proportion of males to females was similar, 51.0% and 49.0% respectively. Sixty-four percent had middle to high education levels, 71.0% were students, and 86.0% were unmarried.

**Table 1:** Participant (n = 364) characteristics*

Age, mean (SD), 15-24 years	18.79 (2.75)
	n(%)
Smoking	81 (22.3)
Gender	
Female	179 (49.2)
Male	185 (50.8)
Education level	
Low	127 (36.2)
Middle-High	224 (63.8)
Labor market position	
Employed	102 (29.1)
Student	249 (70.9)
Marital Status	
Married	52 (14.3)
Unmarried	312 (85.7)
Generation	
First	107 (29.4)
Second	257 (70.6)
Ethnicity	
Moroccan	159 (43.7)
Turkish	205 (56.3)
Religion	
High	111 (31.3)
Low	244 (68.7)
Cultural orientation	
Low	201 (56.1)
High	157 (43.9)
Social contacts	
Low	261 (73.1)
High	96 (26.9)
Depressive symptoms	
No	235 (67.5)
Yes	113 (32.5)
Dep. Symp. mean (SD), (0-60)	13.22 (8.66)

*Numbers do not always add up to the total number of participants due to missing data on those variables.

### Univariable associations with smoking behavior

Table 2 gives the univariable associations with smoking behavior. Significance was tested using Wald chi-square tests with df = 1. The univariable regression analysis showed that the prevalence of current smoking was significantly higher in depressed participants, older adolescents, males, employed young migrants, the Turkish-Dutch group, and those with low perceived importance of religion. Moreover, the relationship between the number of depressive symptoms and the number of cigarettes smoked was investigated using the Pearson product-moment correlation coefficient. There was a significant, positive correlation between two variables [*r *= .22, n = 348, p < .05].

**Table 2:** Univariate associations of sociodemographics, cultural variables, and depressive symptoms with smoking behavior1

	Smokers (%) (n = 81)	Non- smokers (%) (n = 283)	Wald	P-Value	OR (95% CI)
Depressive symptoms					1.00
No	57.1	70.5	4.86	.03	1.79 (1.0 - 3.02)*
Yes	42.9	29.5			
Age, mean (SD)	19.78 (2.47)	18.53 (2.78)	12.40	.00	1.17 (1.07 - 1.28)***
Gender					
Female	34.6	53.4	8.68	.00	**1.00**
Male	65.4	46.6			2.18 (1.29 - 3.68)**
Religion					
High	53.1	73.4	11.59	.00	1.00
Low	46.9	26.6			2.58 (1.53 - 4.37)***
Education level					
Low	32.5	37.2	0.59	.44	1.00
Middle-High	67.5	62.8			0.81 (0.47 - 1.39)
Labor market position			5.06	.02	
Student	53.2	75.9			1.00
Employed	46.8	24.1			2.77 (1.64 - 4.61)*
Marital Status					
Married	16.0	13.8	0.26	.61	1.00
Unmarried	84.0	86.2			0.84 (0.42 - 1.66)
Generation					
First	29.6	29.3	0.00	.96	1.00
Second	70.4	70.7			0.99 (0.57 - 1.69)
Ethnicity					
Moroccan	21.0	50.2	20.05	.00	1.00
Turkish	79.0	49.8			3.79 (2.10 - 6.85)***
Cultural orientation					
Low	61.2	54.7	1.09	.30	1.00
High	38.8	45.3			0.74 (0. 44 - 1.24)
Social contacts					
Low	74.7	72.7	0.13	0.72	1.00
High	25.3	27.7			0.90 (0.51 - 1.60)

^1^All analyses were adjusted for age.

^2^Significance was tested using Wald chi-square tests with df = 1.

^3^p < 0.05, **p < 0.01, *** p < 0.001.

### Multivariable analyses

To assess the association between depressive symptoms and smoking behavior, we conducted a multivariable regression adjusted for all the significant risk factors in the univariable regression analysis. The results are shown in Table 3. The result showing that those who have depressive symptoms smoke more than those with no depressive symptoms was replicated. In addition, current smoking behavior was higher in older participants, males, the Turkish-Dutch group, and in those with fewer religious beliefs than their respective counterparts.

**Table 3:** Multivariable associations of sociodemographics, cultural variables, and depressive symptoms with smoking behavior^1^

	Wald	P-value	OR (95% CI)
**Sociodemographics**			
Age	10.17	0.00	1.19 (1.07 - 1.32)*
Gender			
Female	11.26	0.00	1.00
Male			2.79 (1.51 - 5.16)*
Labor market position			
Student	0.66	0.42	1.00
Employed			1.54 (0.54 - 4.42)
**Cultural variables**			
Ethnicity			
Moroccan	7.53		1.00
Turkish		0.00	2.38 (1.27 - 4.48)*
Religion			
High	12.56	0.00	1.00
Low			2.94 (1.62 - 5.33)*
**Depressive symptoms**			
No	8.43	0.00	1.00
Yes			2.68 (1.45 - 4.97)*

^1^Significance was tested using Wald chi-square tests with df = 1.

*p < 0.001.

## Discussion

We examined the association between depressive symptoms and smoking among young Turkish and Moroccan ethnic minority groups. Depressive symptoms were more prevalent among smokers than among nonsmokers. Twenty-nine percent of subjects with depressive symptoms smoked, compared to 19% of those with no depressive symptoms. Our study also replicated other acknowledged factors related to smoking. Current smoking behavior was more prevalent in those who were older, male, and had paid jobs, a Turkish ethnic background, and perceived religion as less important. When the association between depressive symptoms and smoking was adjusted for these significant risk factors, depressive symptoms still appeared to be related to smoking.

To the best of our knowledge, our study is the first to examine the association between depressive symptoms and smoking among young Turkish and Moroccan ethnic minority groups. A previous study conducted with young Turkish-Dutch migrants focused on Internalizing Problems, namely, Withdrawn, Somatic Complaints, and Anxious/Depressed scales [17]. Internalizing Problems were found to be related to smoking behavior in young Turkish-Dutch migrants. Our study contributed to the limited knowledge on this topic through its specific focus on depressive symptoms and the inclusion of the third-largest ethnic minority group (Moroccan-Dutch) in the Netherlands. Depressive symptoms were assessed using CES-D, which is considered a valid scale for depressive symptoms [21].

There are some limitations to consider in our study. First, smoking status was based on self-reporting, which might have been subject to underreporting smoking status. We aimed to avoid this by allowing the participants to answer the questions on paper instead of during an oral interview. Moreover, we conducted the interviews without the presence of third persons. In addition, we assessed smoking status by one question with a broad time span. Second, our data were based on cross-sectional data, which prevent us from making any causal claims. Research has shown depression as preceding smoking and a consequence of smoking. More prospective studies are needed to establish whether one comes first, or whether there is a third, unrelated factor that predicts the onset of both depression and smoking. Finally, we measured the depressive symptoms by a self-report depression scale, CES-D. Though CES-D is a valid and widely used instrument, it might differ from a clinically administered instrument, and therefore might lead to overestimation in the reports of the symptoms [26].

The significant relationship between depressive symptoms and smoking that we found in adolescents is consistent with previous studies among adults [5]. The longitudinal Stirling County Study found that depressed adults were more likely to start and continue smoking and less likely to quit smoking compared to those who were not depressed [27]. Though this relationship has been studied less in ethnic minorities, Webb and Carey [28] found a relationship between depressive symptoms and smoking behavior among African-Americans.

The relationship between depressive disorders and smoking behavior among young people has not been studied extensively. A number of studies by Rohde and colleagues reported that depressive disorders are both related to smoking initiation and smoking cessation in young adult daily smokers [29]. Consistent with these studies, the results presented here suggest that the presence of depressive symptoms should be assessed in smoking intervention programs for young adults.

In contrast to what we expected, we did not find an interaction between depressive symptoms and gender for smoking behavior. Previous studies indicate that the association between depression and smoking differs between men and women. For example, a study among Chinese-Americans found that depression has a stronger association with smoking among women than among men [30]. However, the association between smoking and depression seems to be similar in our sample. Our results might be caused by the small number of cases, or this relationship may depend on ethnicity. Thus, future studies with different ethnic groups should replicate our analyses.

The association between depressive symptoms and smoking is important for smoking cessation interventions targeted at young people. Smoking behavior may be used as a coping behavior. The possible conditioning between smoking and depression should be investigated in future studies. Indeed, research shows that mental health problems are very common among young adults [7]. In their review, Morrell and Cohen [31] summarized the possible mechanisms that may explain the relationship between depressive symptoms and smoking. Instead of focusing on only one mechanism, the research is in favor of combining biological, psychological, and social mechanisms in the intervention programs. There are no indications that the attention to depression in these interventions should be different for young ethnic minorities.

In line with other populations, we found an association between depression and smoking among Turkish- and Moroccan-Dutch young adults in the Netherlands. The presence of depressive symptoms would probably make smoking interventions difficult. Thus, the significant association between depressive disorders and smoking among young adults deserves further attention. To understand the mechanisms of the association we found, it could be helpful if future research among young ethnic minorities would undertake a more detailed assessment of smoking behavior (including age of smoking initiation, frequency of smoking, duration of smoking, substances smoked, and cultural beliefs about smoking) in relation to depressive symptoms.

Moreover, in addition to attention to depressive symptoms, the relatively high prevalence of smoking in the Turkish population might also be considered. This high prevalence rate has also been found in previous studies [32,33], which indicates that this may be related to beliefs about smoking. Smoking is still normal for Turkish men. Moreover, among women, smoking may be regarded as a status symbol. On the other hand, the parents of young people forbid them to smoke [32,34]. This information should be incorporated into future smoking interventions among young Turkish-Dutch people.

## Conclusion

Our study provides more insight into the association between depressive symptoms and smoking among young adults of non-Western ethnic backgrounds in the Netherlands. We examined whether the depressive symptoms were associated with smoking behavior among young Turkish and Moroccan ethnic minorities in the Netherlands. The results of our study indicate that depressive symptoms are significantly associated with smoking behavior in young Turkish and Moroccan migrants. We also expect that the significant association between depressive symptoms and smoking behavior may be present in other young migrant populations. Thus, further studies with other young migrants would increase the knowledge on the association between depressive symptoms and smoking. In short, the intervention programs for smoking behavior should take depressive symptoms into account for young migrant groups. This could be achieved by assessing depressive symptoms in smoking intervention programs for young adults. We recommend exploring the function of smoking in relation to depressive symptoms, such as reducing negative affect or as an important way of coping with stress.

## Competing interests

The authors declare that they have no competing interests.

## Authors' contributions

CZA participated in the design of the study, performed the statistical analysis, and drafted the manuscript. VN, CA, and KS contributed the concept and critically revised the manuscript. All authors read and approved the final manuscript.

## Funding

This research was supported by the Municipal Health Service Amsterdam (GGD Amsterdam) and the Academic Medical Center in Amsterdam, the Netherlands.
